# The Making of Inlays by the Impression Method

**Published:** 1909-10-15

**Authors:** F. T. Van Woert

**Affiliations:** Brooklyn, N. Y.


### THE MAKING OF INLAYS BY THE IMPRESSION METHOD.

BY F. T. VAN WOERT, M.D.S., BROOKLYN, N. Y.

Read before the Second District Dental Society, March, 1901.

I wish it understood that this paper is not written in defense of the cemented filling nor to convert the skeptic. It is meant to help, if possible, those who recognize its great value, and I hope to make easier the technique necessary for a successful result. The deductions which follow are based upon a practical, clinical experience of over thirty years.

In the beginning my efforts were directed toward the grinding of porcelains to fit cavities on the labial surfaces of the anterior teeth. I have seen within the year a porcelain filling which I inserted between fifteen and sixteen years ago, and am happy to say I found it just as perfect as when it was first inserted. This is but one of many that have stood the test of time, and my greatest regret is that a more practical means of securing such results was not available in the beginning of my professional career.

Previous to the presentation of Dr. Jenkins's porcelain and his method of manipulation, it was a hard struggle and one accompanied by very uncertain results, we have at our command to-day well-tried and scientific methods to assure us of success. It is perfectly natural that this, like all advancements, is to have its opponents and, perhaps, there will be some who will never accept it. I hope not, for their own sake. I am thoroughly convinced that the cemented filling will supplant most of the others, particularly gold foil. If this is true it becomes simply a question of selecting from the various methods the one which promises the best results. And if in the selection there is one which minimizes time and fatigue to patient and operator without sacrificing anything to the ultimate result, it is by all means the one to adopt; and it is for the purpose of helping in this selection that the subject of making inlays by the impression method is presented for your consideration this evening.

REQUISITES FOR SUCCESS WITH THE IMPRESSION METHOD. The essentials necessary for securing an accurate impression of any cavity are: first, suitable trays; second, proper impression material, and, third, a knowledge of its manipulation.

The first requisite, that of trays, I have found can be secured only by making one for each case. I have several forms of those which are for sale at dental depots, but have never been able to secure a satisfactory result with any of them. First, because there are hardly ever two cases alike, and a tray which will give the best results must have its formation to correspond with all the outer lines of the cavity, overlapping them to a certain extent, so that when the impression material is forced into place such surplus as exudes from beneath the tray is in a direct right angle from the cavity margin. As an illustration of this I will pass for your inspection a few prepared cavities and impressions which I hope will make this point clear.

The material for trays which has given me the most satisfaction is sheet platinoid of 32, 34 and 36 gauge, because it has a rigidity, together with more pliability than any other metal that I have been able to find. Another very good quality, while not essential, is that it has a finely finished surface, which at least has the appearance of being clean, and is pleasing to the patient. The bending or shaping of these trays is something which is impossible for me to describe on paper, but I will be glad to demonstrate this after the meeting to anyone who cares to see it.

Manipulation of Impression Compound. The second requisite, the impression material, seems to be a matter of opinion. I have had the pleasure of visiting some of the prominent men in the West and the extreme South within the last month, and find that there are few who agree upon any one make of material, but all agree that one of the forms of so-called modeling compound is the most practical. Personally, I prefer that made by the Detroit Dental Manufacturing Company, because it softens at a lower temperature, sets quicker, and when cold is as hard, if not harder, and gives a very much sharper definition of detail than others which I have tried. It was the faulty manipulation of this material which was the cause of my failure prior to the demonstration which Dr. Taggart gave me at the time of my visit. I had been in the habit of warming it as I would for taking an impression of the mouth, when it should have been prepared with the surface coming in contact with the cavity so soft that it is practically sticky, while that portion connecting with the tray should be quite hard. In other words, the main body of the compound should have enough resistance to force the softer portion into the desired position and retain it until it can be chilled or cooled. This can be accomplished in the following way: After forming the tray, a suitable quantity of compound is heated, the tray held over the flame until it is hot enough for the material to adhere to it, and the compound then pressed into a cone shaped mass with the fingers, and then chilled. The surface of the cone should be held in a small flame, so that it is quickly heated to the point of running and then forced into position, and either compressed air or cold water used for setting it.

I find it a great advantage in large cavities in molars and bicuspids to force between the tray and adjoining tooth the blade of a thin cement spatula to bring up a sharp line at the cervix. This is easily removed after the chilling and facilitates the removal of the impression as well. This is frequently advantageous in approximal cavities of the anterior teeth also.

METHOD OF MAKING AMALGAM MODELS. If we have succeeded in securing an accurate impression, it is only the beginning of a successful ultimate result, and the next procedure, that of making the model, requires as careful consideration and manipulation as any part of the technique. Various materials have been recommended for this purpose, all of which I have given a most careful and impartial trial, and I am forced to the conclusion that there is but one reliable material, and that is a good amalgam. When I say "a good amalgam," I mean one having good edge strength, as little shrinkage as possible and the property of setting quickly, although this is not essential. I use the Standard alloy made after one of Dr. Black's formulas.

First the impression must be imbedded in plaster to a sufficient depth, and with enough body surrounding it to permit of pressing the amalgam well down into the impression. The amalgam is then mixed with enough mercury to make it very plastic, and this is burnished into place with suitable instruments until the impression is filled. Then the excess of mercury can be eliminated by folding a piece of rubber dam several times, and placing it on the amalgam and pressing upon it with the thumb.

The mixing of the amalgam is one of the most important points in the procedure. In my early efforts I tried to fill these impressions as I would a cavity in a tooth, and the force required in burnishing it to place invariably marred the impression which resulted in an imperfect model of the cavity.

ADVANTAGES OF IMPRESSION METHOD. If we succeed in getting an accurate model, a filling made to fit it must fit the cavity which it represents. This being the case, let us consider the advantages to be derived from the impression method. First, we are none of us so perfect in any branch of our art that we are not liable to make mistakes. Second, it is beyond question that we all have many accidents that are just as deplorable as the mistakes we might make, and when such happens in the direct method of making inlays we are obliged to acquaint our patients with the fact that we have erred, or met with a misfortune in the form of an accident, either of which is humiliating to the operator and frequently exasperating to the patient, and, occasionally, to such an extent that the patient loses confidence and seeks services elsewhere.

We will take, for example, porcelain restorations. In the direct method, where the matrix is burnished to the cavity, which, by the way, is a very much more tedious operation than that of taking an impression, we have confronting us the possibility of some distortion in its removal, or, perhaps, in the handling after it has been successfully removed, as well as the possibility of warping in the fusing of the porcelain itself. There is still further the difficulty which arises in many cases of securing a suitable color, or just the proper form of contour, all of which is a large combination of defects which remains to be explained to the patient.

The impression method eliminates all of these difficulties. In the first place, the matrix is secured by swedging the gold into the die with the Brewster press, and the swedged matrix is less likely to change its shape when removed, than the burnished one. The shape of the swedged matrix can be retained by filling it with a hard wax; it is then removed and invested, and later the wax washed out. Should the filling prove a failure, another, or several others, if necessary, can be made without the patient's knowledge, and where the question of color or contour is liable to cause trouble, several fillings, varying from a light to a dark shade, can be made; or if it be a troublesome contour, several of different shapes, so that when the patient presents the suitable filling can be selected without subjecting him to another or several operations and without the unnecessary loss of time to the operator.

Cast-Gold Fillings. The same procedure is applicable with cast-gold inlays, with the exception that the wax filling is fitted to the tooth, as described by Dr. Taggart, omitting the final carving of detail in bite and contour which should be done to the die. If the die is correct the wax filling will go to place without difficulty, but one is surprised to note the little defects in the filling, such as here and there a small point where the wax has not conformed to the sharp edge of the cavity margin. This is due to the lack of resistance at such places, the wax being of one temperature throughout its entire body it is forced by the occlusion from inward out, and on a line with the cavity margin. It may be said that this defect can be remedied by running a hot spatula around the line, but I have found this extremely difficult, particularly at the cervix. It is also claimed that such defects may be corrected by burnishing the gold casting after it has been cemented to place. This has proven just as difficult and unreliable in my hands. And it is a potent point that these difficulties do not exist when cast fillings are made from the impression and amalgam model properly constructed.

It is a fact that many eminently satisfactory results are obtained from the direct method, but that it is less reliable in the hands of the average practitioner is also true. There are many things not strictly related to either method, which play a large part in the success or failure of these operations, and which are liable to be charged against either of them, such as imperfect cavity preparation; faulty occlusion; defective fusing of porcelain; or the careless casting of gold, any one of which would destroy the most perfect work in either of the methods mentioned.

The advantages of the impression method, as I see them, I hope have been set forth clearly, and if there is any one present who can point out a better or more reliable way I am open to conviction and shall be more than gratified.

In conclusion, I want to reiterate the statement made in the beginning of this paper, that the cemented filling will supplant most of the others, particularly gold-foil.—*Items of Interest.*

